# Application of Artificial Neural Networks for Dengue Fever Outbreak Predictions in the Northwest Coast of Yucatan, Mexico and San Juan, Puerto Rico

**DOI:** 10.3390/tropicalmed3010005

**Published:** 2018-01-05

**Authors:** Abdiel E. Laureano-Rosario, Andrew P. Duncan, Pablo A. Mendez-Lazaro, Julian E. Garcia-Rejon, Salvador Gomez-Carro, Jose Farfan-Ale, Dragan A. Savic, Frank E. Muller-Karger

**Affiliations:** 1 Institute for Marine Remote Sensing, University of South Florida, College of Marine Science, 140 7th Avenue South, Saint Petersburg, FL 33701, USA; carib@usf.edu; 2 Centre for Water Systems, University of Exeter, Harrison Building, North Park Road, Exeter EX4 4QF, UK; A.P.Duncan@exeter.ac.uk (A.P.D.); D.Savic@exeter.ac.uk (D.A.S.); 3 Environmental Health Department, Graduate School of Public Health, University of Puerto Rico, Medical Sciences Campus, P.O. Box 365067, San Juan, PR 00936, USA; pablo.mendez1@upr.edu; 4 Centro de Investigaciones Regionales, Lab de Arbovirologia, Unidad Inalámbrica, Universidad Autonoma de Yucatan, Calle 43 No. 613 x Calle 90, Colonia Inalambrica, Merida C.P. 97069, Yucatan, Mexico; julian.garcia@correo.uady.mx (J.E.G.-R.); jafarfan@gmail.com (J.F.-A.); 5 Servicios de Salud de Yucatan, Hospital General Agustin O’Horan Unidad de Vigilancia Epidemiologica, Avenida Itzaes s/n Av. Jacinto Canek, Centro, Merida C.P. 97000, Yucatan, Mexico; sgomezcarro@gmail.com

**Keywords:** nonlinear models, *Aedes aegypti*, *Aedes albopictus*, remote sensing, early warning systems

## Abstract

Modelling dengue fever in endemic areas is important to mitigate and improve vector-borne disease control to reduce outbreaks. This study applied artificial neural networks (ANNs) to predict dengue fever outbreak occurrences in San Juan, Puerto Rico (USA), and in several coastal municipalities of the state of Yucatan, Mexico, based on specific thresholds. The models were trained with 19 years of dengue fever data for Puerto Rico and six years for Mexico. Environmental and demographic data included in the predictive models were sea surface temperature (SST), precipitation, air temperature (i.e., minimum, maximum, and average), humidity, previous dengue cases, and population size. Two models were applied for each study area. One predicted dengue incidence rates based on population at risk (i.e., numbers of people younger than 24 years), and the other on the size of the vulnerable population (i.e., number of people younger than five years and older than 65 years). The predictive power was above 70% for all four model runs. The ANNs were able to successfully model dengue fever outbreak occurrences in both study areas. The variables with the most influence on predicting dengue fever outbreak occurrences for San Juan, Puerto Rico, included population size, previous dengue cases, maximum air temperature, and date. In Yucatan, Mexico, the most important variables were population size, previous dengue cases, minimum air temperature, and date. These models have predictive skills and should help dengue fever mitigation and management to aid specific population segments in the Caribbean region and around the Gulf of Mexico.

## 1. Introduction

Dengue fever is considered a global burden, with more than 500,000 cases reported annually [1,2,3,4]. This vector-borne disease is mostly transmitted by the *Aedes aegypti* mosquitoes, but can also be transmitted by *Aedes albopictus* [1,3]. *Aedes aegypti* are found in tropical/sub-tropical areas, where they have adapted to urbanized environments. This complicates management and mitigation of the organism and the disease [2,5,6,7]. Countries in the Gulf of Mexico and Caribbean have adopted various methods to control spreading of the disease, including mosquito control, monitoring and early warning systems, and educating the population [8,9,10]. The state of Yucatan (Mexico) and the island of Puerto Rico (USA) first reported dengue fever cases in the late 1970s. In successive seasons, a cyclic occurrence and all four serotypes (i.e., DENV-1, DENV-2, DENV-3, and DENV-4) have been reported [9,11,12,13]. Each location reports around 10,000 cases annually [13,14,15,16].

In Yucatan, dengue cases are monitored by the National Epidemiological Surveillance System. The system issues weekly epidemiological reports that track incidence [14]. The Dengue Surveillance System from the USA Centers for Diseases Control and Prevention (CDC) and Puerto Rico’s Department of Health publish similar weekly reports [15]. The phenology of dengue fever cases for both locations is similar, with cases typically increasing in August and September, and decreasing around December and January. This follows the rainy season at both locations [2,15]. In Yucatan, the magnitude of dengue fever epidemics varies, in part due to different serotypes expressing themselves in different years and population susceptibility and movement to and from affected areas [17,18,19,20]. Puerto Rico shows similar patterns [15]. Understanding factors that may lead to an epidemic and some predictive capability are important to design and implement strategies that mitigate incidence [4,21,22,23,24,25,26].

Disease occurrence models can be based on linear and nonlinear approaches that simulate complex relations between short- and long-term (climate) environmental variables and dengue fever incidence [1,27,28]. Linear models are often unable to simulate complex interactions between these factors, and powers tend to be smaller [2]. Nonlinear approaches have generally shown greater power than linear models [29]. For example, Husin et al. [30] predicted dengue fever in Malaysia using a nonlinear model, to help the government fight the disease. A similar study in Singapore used genetic algorithms and support vector machines to predict the number of dengue fever cases [31]. Studies in Thailand, Singapore, and Malaysia have also used artificial neural network (ANN) models to predict dengue fever cases, achieving accuracies greater than 80% [32,33,34]. A similar study in Sri Lanka with ANNs showed a lower accuracy (i.e., 60%) [35]. ANNs are attractive because they generally achieve a higher skill than other types of models [36].

Artificial neural networks use combinations of predictor variables (e.g., environmental factors) to simulate relationships with target variables (e.g., dengue fever outbreak occurrences). These models can be adapted to assimilate data, and this helps improve the functional relationships between climatic factors and dengue fever outbreak occurrences. In our present study, we applied ANNs trained with genetic algorithms to predict dengue fever outbreak occurrences in the state of Yucatan, Mexico, and in San Juan, Puerto Rico. We identified environmental factors that are important in driving dengue fever outbreak occurrences at these different locations. The candidate variables were air surface temperature, sea surface temperature (SST), humidity, precipitation, previous dengue cases, and human population size. Previous dengue cases are defined as those cases that occurred weeks before an outbreak [37]. The model was used to detect occasions when the number of cases would go above a threshold and lead to potential outbreaks. These models were also used to predict dengue fever outbreak occurrences based on population at risk (i.e., younger than 24 years) and vulnerable population (i.e., younger than five years and older than 65 years) [1,38,39,40]. Our results help us to understand dengue fever dynamics and improve epidemiological surveillance and early warning for specific population segments in the Caribbean and Gulf of Mexico.

## 2. Materials and Methods

### 2.1. Study Area

**Figure 1:**
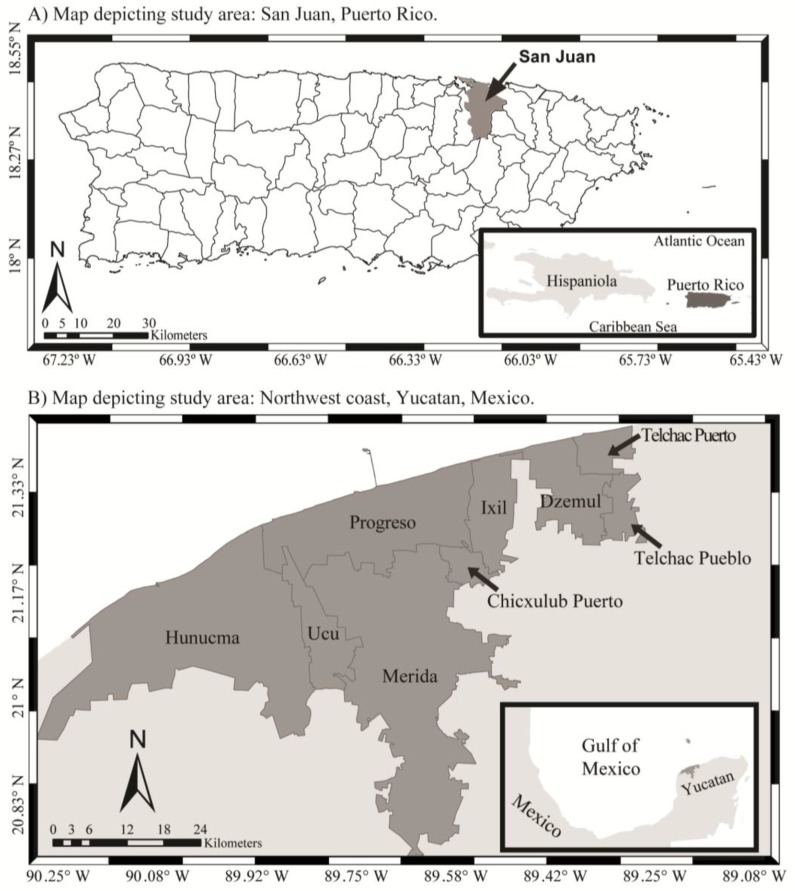
Map of locations of study areas. Maps depicting locations of the study areas in: (**A**) the municipality of San Juan, Puerto Rico; and (**B**) northwest coast of the Yucatan Peninsula, Mexico.

The study areas were the municipality of San Juan in Puerto Rico, USA (17.92° N–18.52° N, 65.62° W–67.28° W), and the northwest coast of the state of Yucatan, Mexico (19.55° N–21.63° N, 87.53° W–90.40° W; Figure 1). San Juan has an average precipitation of ~1800 mm per year, with annual average air surface temperatures of 24–29 °C [41]. Municipalities included for Yucatan State were Chicxulub Pueblo, Dzemul, Hunucma, Ixil, Progreso, Telchac Pueblo, Telchac Puerto, Ucu, and Merida. Yucatan shows higher precipitation between July and October (400–700 mm). The dry season occurs between March and June (0–50 mm). Air temperatures generally range from 36 to 40 °C during the dry season and 30–35 °C during the rainy season [42,43].

### 2.2. Data Sources

#### 2.2.1. Dengue Fever Cases and Demographic Data

Confirmed daily dengue fever data for the northwest coast of the Yucatan Peninsula (*n* = 12,448 daily cases) were obtained from the Universidad Autonoma de Yucatan (2007–2012). Data of confirmed dengue fever cases for San Juan (*n* = 5678 daily cases) were provided by the Dengue Branch of the USA CDC located in Puerto Rico and the Puerto Rico Department of Health, through the University of Puerto Rico, Medical Science Campus. The data included the number of cases per day from 1994 to 2012. The daily incidence data were used to calculate weekly dengue cases for both locations. These were converted to incidence rates (i.e., number of cases per 100,000 inhabitants) using data from Puerto Rico’s Census for San Juan, and using the National Institute of Statistics and Geography for Mexico. Total weekly observations for Puerto Rico were 986, and 310 for Mexico. These cases were further divided into population younger than 24 years, and population younger than five years and older than 65 years old. Total daily cases for Puerto Rico younger than 24 were *n* = 3466; Puerto Rico younger than five and older than 65 years were *n* = 736; Mexico younger than 24 were *n* = 7908; and Mexico younger than five and older than 65 years were *n* = 735.

#### 2.2.2. Environmental Data for San Juan and Yucatan State

Precipitation and minimum and maximum air surface temperatures were obtained from the USA National Oceanic and Atmospheric Administration’s (NOAA) National Centers for Environmental Information (1994–2012) for San Juan. Data for Yucatan from 2007 to 2012 were obtained from the National Water Commission of the state of Yucatan. Weekly means were calculated for these datasets. SST data for both Puerto Rico and Mexico were obtained from the NOAA Advanced Very High Resolution Radiometer (AVHRR; 1 km spatial resolution) satellite-based sensor. SST data were extracted from 1994 to 2012 (Puerto Rico) and 2007 to 2012 (Mexico) using the average of three 3- by 3-pixel boxes located immediately offshore (i.e., coastal area) of the areas of study. These covered a total of 9 km^2^ along the coast. We used Interactive Data Language (IDL; v. 7.2) to extract data.

#### 2.2.3. Data Input and Organization

Weekly dengue incidence rates were log-transformed and used as inputs for the predictive model. Environmental variables used to predict dengue fever in Mexico were: humidity, cumulative one-week precipitation, SST, population size, previous dengue incidence, minimum air temperature, and date. For Puerto Rico, the data included: cumulative four-week precipitation, SST, population size, previous dengue incidence, minimum and maximum air temperature, and date. Significant four-week and one-week cumulative lags for precipitation were identified for Mexico and Puerto Rico, respectively, using Pearson’s correlation analyses [2,43]. Selected predictors were based on previous work done in the study areas that identified these factors as significantly correlated with dengue fever cases [2,15,44].

The models were configured to predict dengue fever outbreak occurrences of population at risk and of the most vulnerable population segments. Mendez-Lazaro et al. [15] identified population at risk in Puerto Rico as people younger than 24 years, and more specifically the most vulnerable to be people younger than five and older than 65 years. These age groups were also used for Mexico, based on dengue fever cases data and previous studies [19,45,46,47]. The weekly data were thus partitioned as: Puerto Rico: population at risk *n* = 986 weekly cases; vulnerable population: *n* = 986 weekly cases. Population at risk in Mexico: *n* = 310 weekly cases; vulnerable population: *n* = 310 weekly cases. These numbers were similar for both population segments per location as data showed dengue fever cases for all age-groups.

### 2.3. Artificial Neural Network Model Setup

#### 2.3.1. Training and Validation

This study implemented the ANN used for the Radar Pluvial flooding Identification for Drainage System (RAPIDS), which was developed to predict flooding in sewer systems by the University of Exeter and was modified to predict dengue fever outbreak occurrences [48,49]. The approach normalizes data (e.g., natural log transformation) to make the weight values more sensitive to changes. The ANNs models used a nondominated sorting genetic algorithm II (NSGA-II) for optimization and training purposes [50,51]; results were validated using the leave-one-out-cross-validation (LOOCV) approach [51,52]. Our objective was to reduce false positives (FP) and false negatives (FN). A weighting factor (‘*a*’) was used within the model to provide a higher relative importance of false positive ratios (FPR) over false negative ratios (FNR) [53]. This value minimized the number of incorrectly predicted passes (i.e., values below thresholds). Different *a*-factor values were tested as part of the testing process, and *a* = 3 was used for Puerto Rico and Mexico. By using this value, we weighted health risks as three times more important. 

The model runs were evaluated for accuracy and predictive power. An accuracy band was calculated based on the percentage of true positives (TP) and true negatives (*TN*) compared to false positives and false negatives (*FP* and *FN*) relative to actual observations. These values change throughout the stages of the models and final values were an overall average based on a confusion matrix (or error matrix). The power of the model to predict these outcomes was based on an F-measure (*FM*; Equation (1)), which provided the importance of *FP* over *FN* using the weighting factor (*a*) discussed above; these values ranged from 0 to 1. The area under the receiving operating characteristic (ROC) curve, or AuC, was also used to test model power, and this was based on TPR and TNR [51]. The ROC curve helped establish the optimal trade-off between FPR and FNR (i.e., FPR = 1 − TPR) [51].

(1) $$FM=\frac{\left(1+a\right)TN}{\left(1+a\right)TN+aFP+FN)}$$

**Figure 2:**
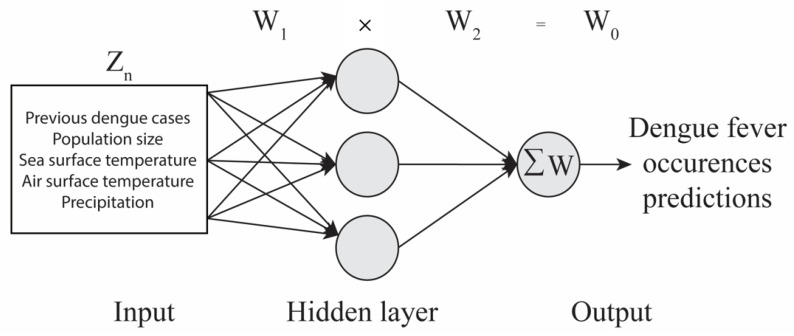
Artificial neural networks (ANNs) schematic. Example of an ANN schematic showing the input layer, hidden layer, and output layer. ANNs calculate weights from variables (Z_n_) in the input to hidden layer (W_1_). The hidden layer then combines these weights and calculates a new set of weights (W_2_). The neural pathway strengths (W_0_) are then calculated, with the equation above the schematic (W_1_ × W_2_ = W_0_), to obtain the strength of influence to predict outcomes, following Duncan et al. [51] and Duncan [56].

In addition to accuracy and model power, the strength of the relationships and relevant influence (i.e., excitatory or inhibitory) between predictors (i.e., environmental factors) and target variable (i.e., dengue fever outbreak occurrences) was assessed. The model weighted in environmental factors and identified those that had the most influence to predict dengue fever outbreak occurrences. The neural pathway strength feature selection (NPSFS) method was used to identify the most relevant inputs, by creating an ensemble of ANNs and comparing the similarities of the weight results (i.e., pathway strength) for the model inputs [51]. Those inputs with the most similarity of pathway strengths (i.e., the strength of relationship) across the whole ensemble of ANNs were selected as the most relevant [51]. First, weights and biases were calculated to understand the relationship between inputs (combined environmental factors) and outputs (dengue fever outbreak occurrences) through NPSFS [51,54,55]. The ANN models processed data values unidirectional from inputs towards outputs, creating these sets of weights and biases [51]. The weights were calculated for the hidden layers (W_1_) and for the output layer (W_2_; Figure 2). The neural pathway strengths (W_0_) of each input were calculated after the completion of the training using NSGA-II, employing the NPSFS methodology (i.e., matrix math of the ANNs hidden layer weights matrix (W_1_) and ANN output layer weights vector (W_2_)) to identify the most influential factors [47]. Each of the members of the ensemble is trained on a similar but different subset of the full training data set. As a result, the weights obtained in each ANN will have different values. The net effect of each input was analyzed by NPSFS, by multiplying W_1_ to W_2_, as shown above. Therefore, for a single output ANN, the result is a vector that specifies the combined pathway strength of each input on the output (neglecting non-linearity of activation functions). These weights were then combined through the combined neural pathway strength analysis (CNPSA) by looking at the spread of the pathway strengths for each input, where we identified if the majority of these treated the given input in the same sense, excitatory or inhibitory [54,55]. If so, we say that the input is relevant to predict potential dengue fever outbreak occurrences.

These weights were optimized using the crossover and mutation rates, incorporated by NSGA-II, during the training period [48]. The NSGA-II crossover and mutation rate factors helped differentiate each new weight generation from the parent weight generation for predictive purposes [51]. Different crossover and mutation rates were tested iteratively. The optimal values to predict dengue fever outbreak occurrences were a 0.1 crossover rate and 0.2 mutation rate for both locations. The minimization of cost (i.e., cost function) was based on false positive rates and false negative rates. These were derived by the ROC using the minimum Euclidean distance to the ideal true positive ratio equal to one [56]. These were used for optimization by NSGA-II to assess the quality of solutions [51,56].

Training, validation, and testing of dengue fever outbreak occurrences was achieved by dividing the data from Puerto Rico and Mexico into different epochs (i.e., years). Model sample sizes were identified as follows: Puerto Rico less than 24 years (N_PR24_), Puerto Rico younger than five and older than 65 years (N_PR5-65_), Mexico younger than 24 (N_MX24_), and Mexico younger than five and older than 65 years (N_MX5-65_). For Puerto Rico, the first ten epochs of data were divided into eight for training and two for validation. The validations were done every fifth epoch. The following seven epochs had six for training and one for validating. Validation was done every third epoch. The last two epochs of data were used for testing. Therefore, we had 14 epochs for training (N_PR24_ = 1950 daily cases; N_PR5-65_ = 437 daily cases), three epochs for validating (N_PR24_ = 609 daily cases; N_PR5-65_ = 140 daily cases), and two epochs for testing (N_PR24_ = 907 daily cases; N_PR5-65_ = 159 daily cases), for a total of 19 epochs. 

Due to the shorter time series, some of the years/epochs for the Yucatan dataset were divided into two (i.e., 26 weeks/epochs each), totaling 12 epochs. The first ten epochs were divided into eight training epochs and two validating epochs; this was done by training three consecutive epochs and using the fourth as validation. The last two of those ten epochs were used as training, and epochs 11 and 12 were testing epochs. There was a total of six epochs for training (N_MX24_ = 2169 daily cases; N_MX5-65_ = 187 daily cases), two epochs for validating (N_MX24_ = 1643 daily cases; N_MX5-65_ = 89 daily cases), and two epochs for testing (N_MX24_ = 4096 daily cases; N_MX5-65_ = 459 daily cases). These divisions ensured that data for training and validation were different from those used for the final testing of predictions.

#### 2.3.2. Thresholds to Identify and Predict Potential Dengue Fever Outbreaks

**Figure 3:**
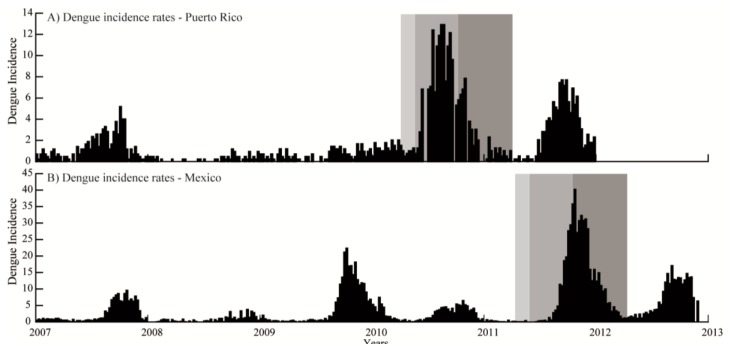
Dengue fever incidence rates distributions for San Juan, Puerto Rico and the northwest region of Yucatan, Mexico. Dengue incidence rates per 100,000 inhabitants from 2007 to 2012 for (**A**) San Juan, Puerto Rico; and (**B**) northwest coast of Yucatan, Mexico. Data only includes these years to show patterns and epidemic years, which for both locations were 2007 and 2011. The three shaded gray boxes represent the pre-epidemic (weeks 10–20), epidemic (weeks 21–40), and post-epidemic (weeks 41–49) periods.

Specific thresholds were identified for Mexico and Puerto Rico to predict potential outbreaks based on dengue fever outbreak occurrences. The number of cases was divided into three periods for a full year (52 epidemiological weeks): pre-epidemic (weeks 10–20); epidemic (weeks 21–40); and post-epidemic (weeks 41–49) for Puerto Rico, based on the work of Mendez-Lazaro et al. [15]. This same distribution was used for Mexico, based on weekly dengue cases data from 2007 to 2012 (Figure 3) [2]. The periods were identified based on the distribution of cases, where the pre- and post-epidemic periods showed a slow increase and decrease in number of cases, respectively, and the epidemic period was when the majority of the cases were observed. The average number of cases during those periods was then calculated.

To provide an epidemic threshold for the model, the 75th percentile of all weekly cases per year was calculated for each period and age-distributions. The 75th percentile for the threshold has been used and identified by previous studies as the cutoff for threshold selection regarding infectious diseases [57,58,59]. Dengue fever epidemics were identified as three or more suspected cases (per 1000 individuals) for two consecutive weeks [15]. These threshold numbers represent the number of cases per week. The following thresholds were obtained and used in the models. For Puerto Rico, population younger than 24 years: 2 (pre-epidemic), 6 (epidemic), and 5 (post-epidemic); and population younger than five and older than 65 years: 1 (pre-epidemic), 2 (epidemic), 1 (post-epidemic). For Mexico: population younger than 24 years: 2 (pre-epidemic), 33 (epidemic), and 13 (post-epidemic); and for population younger than five and older than 65 years: 1 (pre-epidemic), 6 (epidemic), and 4 (post-epidemic). The models compared the observed and predicted values to each of these thresholds to identify them as a ‘no outbreak period’ (i.e., value below threshold) or a ‘potential outbreak period’ (i.e., value above thresholds). These models were run as categorical models; therefore, the results were interpreted based on the influence, and magnitude, of inputs to predict a potential outbreak based on the specific thresholds mentioned above. Therefore, the results are shown as inputs having an (inhibitory or excitatory) influence on outputs in terms of outputs crossing the set thresholds.

## 3. Results

### 3.1. Model Accuracy

The accuracies of the predictions for both locations were similar, with predictions for the ‘no outbreak periods’ having a higher accuracy compared to predictions for the ‘potential outbreak periods’. Model accuracy bands for San Juan, Puerto Rico were 47% for the population at risk model (i.e., younger than 24 years), and 58% for the vulnerable population model (i.e., younger than five and older than 65 years). More specifically, the 47% accuracy band for the population at risk model represented a prediction of 19% of the ‘potential outbreak periods’ and 28% of the ‘no outbreak periods’. The model representing the most vulnerable population, with an accuracy band of 58%, represented a prediction of 17% of the ‘potential outbreak periods’ and 41% of the ‘no outbreaks periods’. These models showed a higher accuracy band for periods of ‘no outbreaks’ compared to ‘potential outbreak periods’.

A similar pattern was observed for models in Mexico. The population at risk model in Mexico showed an accuracy of 51%, which represented an accurate prediction of 12% of ‘potential outbreaks period’ and 39% of predicted ‘no outbreaks periods’. The most vulnerable population model showed a 66% accuracy band, representing 5% of predicted ‘potential outbreak periods’ and 61% of ‘no outbreak periods’. In both instances, the four models showed a higher accuracy for the ‘no outbreak period’ compared to ‘potential outbreak period’, and the accuracies were higher for the most vulnerable population models compared to the population at risk models.

### 3.2. Evaluation of ANNs Model Predictive Power Based on F-Measure and ROC Curve

**Figure 4:**
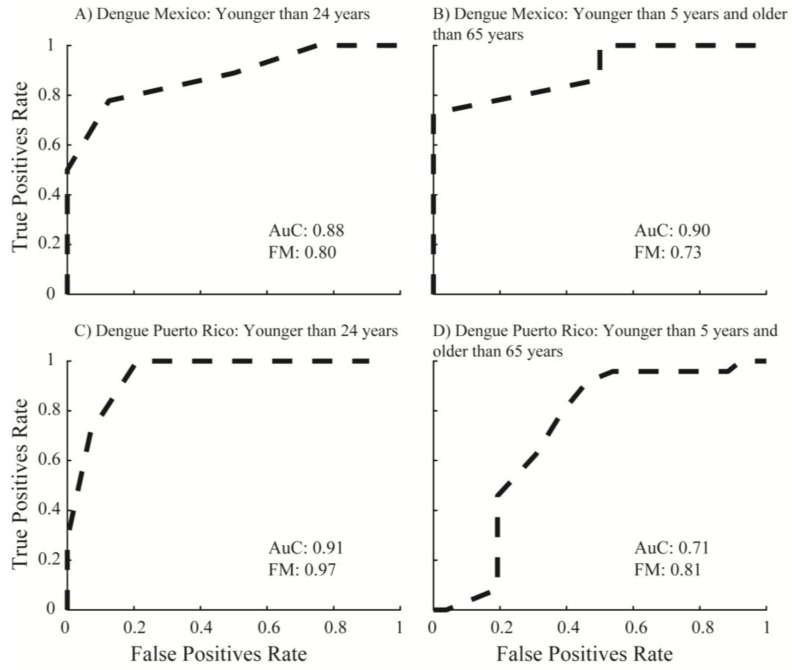
Performance of model to predict dengue fever outbreak occurrences. Graphs show the receiver operating characteristic (ROC) curve results of the four artificial neural network models for San Juan and Mexico. Dashed lines are the calculated true positive ratios (TPR) and false positive ratios (FPR) for all four models. The areas under the curves (AuC) were calculated based on TPR/FPR ratios and F-measures (FM) describe the importance of false positives over false negatives. These AuC and FM values show models’ power [55]. AuC and FM were calculated for: (**A**) population younger than 24 years, Mexico; (**B**) population younger than five years and older than 65 years, Mexico; (**C**) population younger than 24 years, Puerto Rico; and (**D**) population younger than five years and older than 65 years, Puerto Rico.

The overall power for the four model runs was above 70%. This was based on ROC curves and FM. ROC curves were calculated for all four model runs (Figure 4A–D) using the weights (W_0_) calculated by the ANN ensembles. The population at risk model for San Juan showed an FM of 0.97 and an AuC of 0.91. The most vulnerable population model showed an FM of 0.71 and an AuC of 0.81. Overall, these models showed a predictive power greater than 90% and 70% for San Juan. Conversely, the population at risk model showed a higher predictive power compared to the most vulnerable population model.

Model runs for Yucatan showed similar results as those for San Juan, with the overall power above 70 percent. The population at risk model showed an FM of 0.80 and an AuC of 0.88. The most vulnerable population model showed an FM of 0.73 and AuC of 0.90. However, the overall power for the population at risk model was higher and numbers were closer compared to the power obtained by the most vulnerable population model; thus, these results are consistent with those observed in Puerto Rico. A series of baseline multiple linear regression (MLR) models were also evaluated. The results showed that ANN models had a higher statistical power compared to MLR models using the same data approach (Supplementary Materials, Tables S1–S4).

### 3.3. Environmental Factors Relevant for Dengue Fever Outbreak Occurrences Predictions

**Figure 5:**
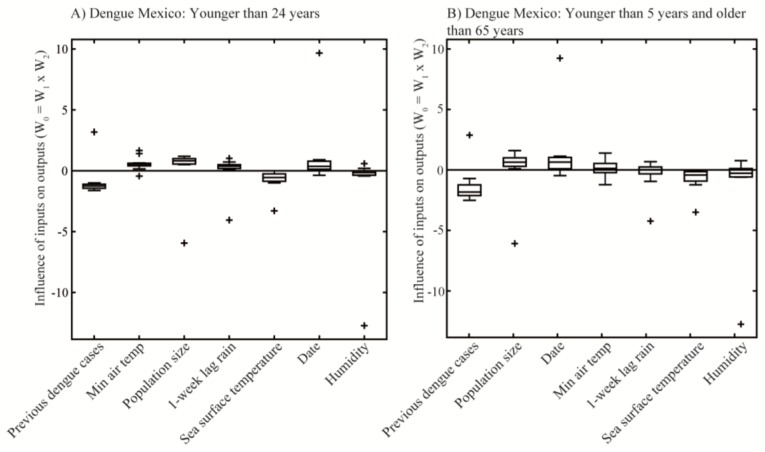

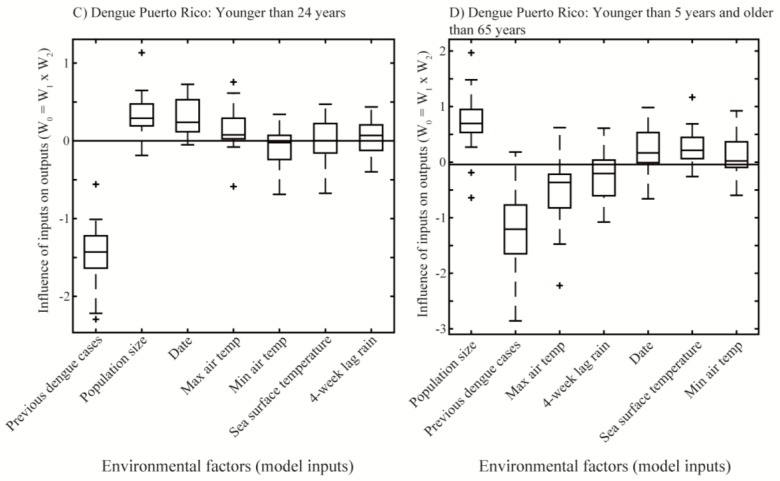
Distribution of environmental factors weights in the ANNs to predict dengue fever outbreak occurrences in Mexico and Puerto Rico. The box and whiskers plots show the influence of each environmental factor on dengue fever outbreak occurrences predictions based on weights distributions (W_0_) calculated by the ANNs’ models. Boxes show the distribution of weights, lines inside the boxes are the mean values of weights. Zero lines represent no relevance for predicting dengue fever outbreak occurrences. These weights are shown in order of importance for: (**A**) population younger than 24 years, Mexico; (**B**) population younger than five years and older than 65 years, Mexico; (**C**) population younger than 24 years, Puerto Rico; and (**D**) population younger than five years and older than 65 years, Puerto Rico.

The models result showed that both environmental factors and demographic variables had an important influence on dengue fever predictions, based on weights calculated through the NPSFS method. These weights (W_0_) represented the strength of the relationship between inputs and outputs, and those variables identified as the most influential had values different than zero. Therefore, those parameters with weights equal to zero were those having no influence on predicting dengue fever outbreak occurrences for Mexico and Puerto Rico. Furthermore, these environmental factors were identified as having an excitatory (represented by positive weights) or inhibitory (represented by negative weights) influence. For example, Figure 5 shows previous dengue cases as having an inhibitory influence on predicting dengue fever outbreak occurrences. This means that, overall, previous dengue cases inhibit dengue fever outbreak occurrences to cross the set thresholds (i.e., pass). Similarly, population size shows an excitatory influence, meaning that this variable stimulates dengue fever outbreak occurrences to cross the outbreak threshold (i.e., fail).

Weights with the most overall influence identified by ANNs in Mexico for the population at risk model were previous dengue cases, minimum air temperature, population size, cumulative one-week lag precipitation, and SST (Figure 5A). However, for the most vulnerable population model, previous dengue cases and population size were the most influential, and other factors only slightly influenced dengue fever outbreak occurrences (Figure 5B). The model runs for Puerto Rico showed that the most influential parameters overall for dengue fever were previous dengue cases, population size, date, and maximum air temperature for the population at risk model (Figure 5C). The model for the most vulnerable population showed population size, previous dengue cases, and maximum temperature as the most influential factors (Figure 5D). The models for Puerto Rico showed a wider spread of weights compared to those for Mexico. Overall, all four model runs had previous dengue fever and population size as the two variables with the most influence on predicting dengue fever in both study areas.

## 4. Discussion

### 4.1. Most Relevant Environmental and Social Factors Influencing Dengue Fever Outbreak Occurrences

Dengue fever cases are influenced by a series of environmental and demographic parameters [60,61,62]. Precipitation, air temperatures, and social factors (i.e., previous dengue cases and population size) were the most influential to predict dengue fever outbreak occurrences in both the Yucatan and San Juan. These factors influence dengue fever elsewhere [2,15,63]. Precipitation and temperatures (i.e., maximum and minimum temperatures) are related to the timing of the mosquitoes’ development and virus replication. Dengue fever outbreak occurrences follow precipitation patterns as these provide breeding sites. Warmer temperatures also reduce the developing time of mosquitoes and help increase their densities [3,64,65]. 

The most influential inputs for predicting dengue fever were those related to demographic changes. Overall, the most relevant factors in predicting dengue fever outbreak occurrences in Yucatan and San Juan were population size, previous dengue incidence, and air temperatures. Changes in population is a key factor influencing virus spreading and further peaks in cases [17,19]. These factors have been demonstrated as drivers in other regions [2,15,44,63]. The relationship between population changes and an increase in dengue fever cases can be due to more people being susceptible to dengue fever (e.g., had never been exposed to the virus; [13,66]).

The second demographic parameter identified as most influential was ‘previous dengue cases’. While this can be affected by changes in population size, this factor could be lower because of increased immunity with time [67,68]. Nevertheless, decreased immunity due to other factors like hygiene and age, as well as shifts in serotypes, can lead to peaks in dengue fever outbreak occurrences due to secondary infections [69,70].

Air temperatures showed a significant influence on predicting dengue fever outbreak occurrences in both study areas. An increase in minimum air temperature can promote mosquito development [2,3], but as it becomes warmer than normal, their development slows down or is inhibited [68]. Increased air temperature is highly correlated with increased mosquito bites due to the animal’s energy demands, leading to higher probabilities of humans becoming infected [71]. Only the model for population at risk for Yucatan showed SST as slightly influential on predicting dengue fever outbreak occurrences, so this factor was not very important relative to those mentioned above for these coastal regions. Lastly, date was also identified as a significant factor by the models. This is related to the cyclic pattern that dengue fever cases show across the Americas. Clearly, there is a correlation and lack of independence between date and seasonality of precipitation patterns [72,73,74,75].

### 4.2. ANNs Model Performance on Predicting Dengue Fever in Mexico and Puerto Rico

The overall power of 70% was obtained through the combination of these variables. This builds upon previous work, where ANN modeling power ranged from 60 to 80% [32,33,34,35]. These power results were also higher compared to multiple linear regression models. By modeling two population segments, those considered vulnerable and those considered at risk, these models were able to identify specific variables that were most influential to understand dengue dynamics depending on susceptibility. The main differences seen in power and accuracy among these models could be due to those younger than 24 years old being at risk due to social mobility (e.g., work, school, home), and those considered most vulnerable could be due to biological conditions such as leakage of plasma (e.g., those less than five years old having a lower threshold for fluid escape from intravascular to extravascular space; [76]) and chronic degenerative diseases (i.e., greater than 65 years old; [77]). Therefore, models identifying demographics as the most influential could be due to: (1) primary infections based on exposure (i.e., population susceptibility; [78]); (2) secondary infections [79]; (3) geographic locations (e.g., houses without protection, such as windows and doors screens; [2,80]; and (4) public services (e.g., trash pick-up, drainage cleaning; [79,80]). Similarly, simultaneous circulation of more than one serotype can increase peaks in cases [19,77,79].

The power calculated by the ANN models, through AuC and FM, accounted for the ratios of true positives and true negatives compared to false positives and false negatives, as shown in Equation (1), and was improved by the weighting factor. On the other hand, the accuracy band only looked at the predicted values individually compared to the original values, where percentages might be affected by the number of pass/fail in the original observations. Therefore, we see a difference between accuracy and power due to the methodology, where the models’ powers were based on the rate and capability of them to predict potential dengue fever outbreaks. Dividing the data into three periods to predict dengue fever outbreak occurrences (i.e., pre-epidemic, epidemic, and post-epidemic) could have also influenced the outcomes of the models. The magnitudes of cases during these periods changed annually. These differences across years are influenced by those factors discussed above (e.g., social factors and epidemiology). Thresholds used for this study were identified using data available for Puerto Rico and Mexico during those specific timeframes. Including longer, or shorter, datasets can influence these thresholds depending on dengue fever dynamics, as these tend to be location specific [81,82].

### 4.3. Study Limitations and Future Work

There were many factors that were not considered in defining the predictive capacity of the ANN models. This includes population susceptibility to dengue fever (e.g., serotypes, population movement). To be able to accurately identify risk and vulnerability to dengue fever outbreak occurrences, the serotype(s) circulating must be known [13,22,83,84,85]. When there is a shift in serotypes, those who had not been previously exposed to the new one become more vulnerable, leading to an increased incidence of dengue fever [66,67]. This is also observed in the different peaks per years, where environmental variables are not the only factors influencing these strengths, thus affecting the accuracy of these models.

Changes in population segments considered at risk and vulnerable may also lead to differences. People that had never been exposed to dengue fever can be part of one of these two population segments, and this was not considered due to data limitations. The thresholds used for the three periods that the data were divided into could have affected the prediction capability of the models and future studies might need to consider different thresholds according to vulnerable population, populations at risks, and geographic locations, as well as circulating serotypes. Lastly, Puerto Rico had 19 years of data, whereas Mexico had six years; however, both study areas provided equivalent results. Future studies should consider expanding these time series to better understand temporal and spatial differences across dengue fever endemic areas, as well as applying ANNs in this area to predict coming seasons.

## 5. Conclusions

Modelling dengue fever using environmental and demographic factors with a nonlinear neural network approach can help predict dengue fever incidence rates in Mexico and Puerto Rico with a power greater than 70 percent. Four model runs, two for population at risk and two most vulnerable population in Yucatan, Mexico and San Juan, Puerto Rico, identified precipitation, population size, air temperature, previous dengue cases, and date as the most influential factors that predict dengue fever outbreak occurrences. Demographic factors, including population size and previous dengue cases, were of most importance. Understanding behavior of the population and education programs can help improve the effectiveness of early warning systems in this region and mitigate the disease. Further studies are needed to incorporate vector and dengue fever virus dynamics into models, as these can help improve the skill of simulations and understand similar diseases that depend on climate and environmental changes.

## Supplementary Materials

The following are available online at http://www.mdpi.com/2414-6366/3/1/5/s1, Table S1. Mexico multiple linear regression model for population younger than 24 years old. Bold values are significant with 95% certainty (α = 0.05). Parametric and non-parametric *p*-values are included. Power of these models (*r*^2^) are shown above the table, Table S2. Mexico multiple linear regression model for population younger than five and older than 65 years old. Bold values are significant with 95% certainty (α = 0.05). Parametric and non-parametric *p*-values are included. Power of these models (*r*^2^) are shown above the table, Table S3. Puerto Rico multiple linear regression model for population younger than 24 years old. Bold values are significant with 95% certainty (α = 0.05). Parametric and non-parametric *p*-values are included. Power of these models (*r*^2^) are shown above the table, Table S4. Puerto Rico multiple linear regression model for population younger than five and older than 65 years old. Bold values are significant with 95% certainty (α = 0.05). Parametric and non-parametric *p*-values are included. Power of these models (*r*^2^) are shown above of the table.
