# Supplementary material for: Application of Artificial Neural Networks for Dengue Fever Outbreak Predictions in the Northwest Coast of Yucatan, Mexico and San Juan, Puerto Rico
Source: Trop Med Infect Dis. 2018 Jan 5;3(1):5. doi: 10.3390/tropicalmed3010005 (PMC6136605; doi:10.3390/tropicalmed3010005)
Supplement: Supplementary file 1 [file tropicalmed-03-00005-s001.pdf]

Application of Artificial Neural Networks for Dengue Fever Outbreak Predictions in the Northwest Coast of Yucatan, Mexico and San Juan, Puerto Rico

Abdiel E. Laureano-Rosario, Andrew P. Duncan, Pablo A. Mendez-Lazaro, Julian E. Garcia-Rejon, Salvador Gomez-Carro, Jose Farfan-Ale, Dragan A. Savic and Frank E. Muller-Karger

Table S1. Mexico multiple linear regression model for population younger than 24 years old. Bold values are significant with 95% certainty (α = 0.05). Parametric and non-parametric p-values are included. Power of these models (r^2) are shown above the table.

Mexico younger than 24 years (r^2 = **0.85**)

Variable	b	t-stat	Parametric p-value	Non- parametric p-value
Date	-327.54	-1.39	0.166	0.102
Humidity	1.02	3.01	**0.003**	**0.002**
SST	1.11	1.51	0.132	0.114
Precipitation 1-week lag	0.07	3.55	**0.000**	**0.002**
Population	-30.08	-1.75	0.081	**0.018**
Minimum air temperature	1.16	3.67	**0.000**	**0.002**
Previous dengue cases	5.46	24.97	**0.000**	**0.002**

Table S2. Mexico multiple linear regression model for population younger than 5 and older than 65 years old. Bold values are significant with 95% certainty (α = 0.05). Parametric and non-parametric p-values are included. Power of these models (r^2) are shown above the table.

Mexico younger than 5 and older than 65 years (r^2 = **0.84**)

Variable	b	t-stat	Parametric p-value	Non-parametric p-value
Date	-79.89	-0.43	0.668	0.654
Humidity	0.44	1.64	0.103	0.220
SST	0.19	0.33	0.743	0.612
Precipitation 1-week lag	0.04	2.97	**0.003**	**0.040**
Population	-11.35	-0.84	0.404	0.364
Minimum air temperature	0.58	2.33	**0.021**	**0.038**
Previous dengue cases	10.37	26.36	**0.000**	**0.002**

Table S3. Puerto Rico multiple linear regression model for population younger than 24 years old. Bold values are significant with 95% certainty (α = 0.05). Parametric and non-parametric p-values are included. Power of these models (r^2) are shown above the table.

Puerto Rico younger than 24 years (**r^2 = 0.74**)

Variable	b	t-stat	Parametric p-value	Non-parametric p-value
Date	-3.62	-2.95	**0.003**	**0.002**
SST	0.07	1.92	0.055	**0.046**
Precipitation	0.00	-1.13	0.258	0.282
Population	-0.19	-6.34	**0.000**	**0.002**
Minimum air temperature	0.03	0.84	0.400	0.370
Maximum air temperature	-0.03	-0.80	0.425	0.394
Previous dengue cases	0.05	41.94	**0.000**	**0.002**

Table S4. Puerto Rico multiple linear regression model for population younger than 5 and older than 65 years old. Bold values are significant with 95% certainty (α = 0.05). Parametric and non-parametric p-values are included. Power of these models (r^2) are shown above the table.

Puerto Rico younger than 5 and older than 65 years (**r^2 = 0.33**)

Variable	b	t-stat	Parametric p-value	Non-parametric p-value
Date	-42.24	-2.21	**0.027**	**0.030**
SST	0.05	0.09	0.928	0.976
Precipitation	0.01	0.42	0.678	0.654
Population	-1.60	-3.37	**0.001**	**0.002**
Minimum air temperature	1.70	2.88	**0.004**	**0.012**
Maximum air temperature	-0.19	-0.31	0.756	0.810
Previous dengue cases	0.38	16.04	**0.000**	**0.002**
